# Visualization of the Crossroads between a Nascent Infection Thread and the First Cell Division Event in *Phaseolus vulgaris* Nodulation

**DOI:** 10.3390/ijms23095267

**Published:** 2022-05-09

**Authors:** Elizabeth Monroy-Morales, Raúl Dávila-Delgado, Emmanuel Ayala-Guzmán, Alicia Gamboa-deBuen, Rosana Sánchez-López

**Affiliations:** 1 Departamento de Biología Molecular de Plantas, Instituto de Biotecnología, Universidad Nacional Autónoma de México, Avenida Universidad 2001, Colonia Chamilpa, Cuernavaca 62210, Mexico; elizabeth.monroy@ibt.unam.mx (E.M.-M.); raul.davila@ibt.unam.mx (R.D.-D.); tepoz_penta@hotmail.com (E.A.-G.); 2 Instituto de Ecología, Universidad Nacional Autónoma de México, Ciudad de México 04510, Mexico; agamboa@ecologia.unam.mx

**Keywords:** cell division, cell plate, cytokinesis, infection thread, nodulation, nucleus, *Phaseolus vulgaris*, rhizobia, KNOLLE

## Abstract

The development of a symbiotic nitrogen-fixing nodule in legumes involves infection and organogenesis. Infection begins when rhizobia enter a root hair through an inward structure, the infection thread (IT), which guides the bacteria towards the cortical tissue. Concurrently, organogenesis takes place by inducing cortical cell division (CCD) at the infection site. Genetic analysis showed that both events are well-coordinated; however, the dynamics connecting them remain to be elucidated. To visualize the crossroads between IT and CCD, we benefited from the fact that, in *Phaseolus vulgaris* nodulation, where the first division occurs in subepidermal cortical cells located underneath the infection site, we traced a *Rhizobium etli* strain expressing DsRed, the plant cytokinesis marker YFP-*Pv*KNOLLE, a nuclear stain and cell wall auto-fluorescence. We found that the IT exits the root hair to penetrate an underlying subepidermal cortical (S-E) cell when it is concluding cytokinesis.

## 1. Introduction

Cell division is crucial for the legume:rhizobia nodulation, a symbiotic process characterized by the development of a new lateral organ on the plant root, an N2-fixing nodule. Nodules are composed of the peripheral tissue, which includes the vascular system and the central tissue or infected zone, where the nitrogen reduction takes place. Two types of nodules exist, indeterminate and determinate [1,2]. Indeterminate nodules are developed in the legumes *Medicago truncatula*, *M. sativa* and *Pisum sativum*, among others, and are defined by an oval shape and a long-lived or persistent meristem, in which the initial cortical cell division (CCD) takes place in the inner most cortical and pericycle cells opposite the protoxylem poles [3]. The mature nodule presents a central tissue organized in gradient zones (I, meristematic; II, invasion zone; III, fixation zone’ IV, senescent zone; and V, saprophytic zone [2]). In contrast, determinate nodules, formed in *Glycine max*, *Phaseolus vulgaris* and *Lotus japonicus*, have a round shape and present limited mitotic activity; in *P. vulgaris*, CCD begins in the outermost C1 cortical cell [4], whereas in *L. japonicus,* it corresponds to cell layer C3 [4]. The central tissue is a mixture of infected and uninfected cells [2,5].

It is noteworthy that, irrespective of their origin and tissue organization, both nodule types share a basic developmental program that comprises two linked and tightly regulated processes: infection and organogenesis [6,7]. Epidermal infection is triggered by the exchange of molecular signals, namely, root flavonoids and rhizobial lipochito-oligosaccharides (Nod factors, NF). The NF signal is decoded in the root hair when the NF are perceived by specific LysM receptor-like kinases, which in turn activates a signaling cascade [6,8]. The responsive root hair curls, trapping the rhizobia in an infection chamber, where bacteria form a microcolony. At this site, the cell wall and plasma membrane invaginate to form an inward, transcellular tunnel-like structure, known as infection thread (IT), which mediates the entry and proliferation of rhizobia [9,10]. The IT growth is anticlinal to the root surface and presents branching episodes. Eventually, the IT elongates and exits the root hair to infect the root cortical zone to invade the neighboring cells. Concomitant to the IT development, cortical cells become mitotically active. Initial CCDs are always anticlinal. An active cell proliferation gives rise to a nodule primordium. The peripheral post-meristematic cells differentiate to form the nodule cortex and the pro-vascular traces. At the young nodule stage, IT invades the central zone to further release the bacteria into the intracellular environment, the symbiosome [1,2]. In this membrane-bound *quasi*-organelle, rhizobia differentiate into bacteroids that express the nitrogenase complex, responsible for the reduction of molecular nitrogen (N2) to supply the legume with bioavailable nitrogen. In contrast, the rhizobia obtain di-carbon compounds, as energy source [11].

Certainly, nodulation is a complex process that recruits part of the lateral root formation and the plant hormone regulatory programs [8,12,13,14,15,16,17,18], as it was revealed by the identification of genes essential for the development of the IT and nodule primordium, as well as by the nodule organogenesis. For the characterization of a variety of legume mutants deficient in nodulation, the phenotypic analysis of gene-specific downregulation/overexpression and transcriptomic analysis have contributed significantly to our current understanding of the IT initiation and progression processes, alongside the cell cycle reactivation in cortical cells at the pole of infection [5,8,16,18,19,20]. The list of genes related to the IT formation process includes receptor-like kinases (NFR5/NFR1/NFP/LYK3 and SYMRK/DMI2/NORK; [21,22,23,24,25]), small GTPases (e.g., ROP3, ROP6, ROP10 and RabA2; [26,27,28,29,30,31]), endocytosis and exocytosis markers (e.g., CHC and VAMP721e [27,32]), cell wall enzymes (e.g., Pectate-Lyase [33]), cytoskeletal proteins (e.g., flotillin, components of SCAR/WAVE and SCARN [34,35,36,37,38,39,40]), E3 ubiquitin ligases (e.g., CERBERUS, SINA and PUB [41,42,43]), transcription factors (e.g., NSP1/NSP2, ERF, ERN1, CYCLOPS/IPD3, NF-Y family members and NIN, a key regulator of the nodulation process [6,44,45,46,47,48,49,50,51]). Interestingly, some of those transcription factors are part of a complex transcriptional network that regulates both epidermal and cortical infection, and promotes nodule organogenesis, i.e., NIN, NSP1 and NSP2, as well as AP2/ERF [32,40,49,52,53]. CCD at early stages of nodulation is controlled by genes related to the cell cycle regulatory machinery and those associated to hormone signaling programs [14,19,20,53,54,55,56,57,58,59,60,61,62]. The first insights into this direction were obtained from early experiments assessing the mitogenic effect of NF on uninoculated alfalfa roots. Inner CCDs were observed in treated roots, and in situ hybridization confirmed the induced expression of cyclin B (cyc2) and cyclin-dependent kinase 2 (cdc2) [63]. Similar results were observed by Roudier et al. [59], when characterizing the gene Medsa;cycA2;2. In the same line, CCD was induced when a pea root-cortex explant was treated with auxin and cytokinin [64]. Moreover, the auxin responsiveness in rhizobium-induced cell division was confirmed by tracing the activity of auxin-responsive promoters (using *GH3*::*GUS*, *DR5*::*GUS* or *DR5*::*GFP* reporters), which were detected at the first division event at the inner or the outer cortex of *P. sativum* or *L. japonicus* infected roots, respectively [58,65]. Further reports corroborated that auxin efflux and influx, auxin transport inhibitors, auxin response factors and microRNA160 play a central role in rhizobial infection, cell cycle control and differentiation of vascular tissue in the nodulation process [17,19,59,66]. The phenotypic characterization of the *L. japonicus* gain-of-function and loss-of-function mutants *snf2*/*lhk1* and *hit1* and *M. truncatula* CRE1-RNAi roots [60,62,67] paved the way for deciphering the functions of the cytokinin signaling pathway, which includes genes involved in the biosynthesis and degradation of cytokinin, the participation of responsive regulators and transcription (reviewed in [16]), as part of the network controlling nodulation.

Notwithstanding the relevance of the molecular mechanisms discussed above, the cellular relationship between infection and CCD has yet to be established. It is also important to keep in mind that the nodule organogenesis begins with the onset of a new meristem, which implies that progenitor cortical and pericycle cells transit from a differentiated state towards the acquisition of a proliferative competence, a process that remains to be fully elucidated. Moreover, to invade the nodule primordium cells, the IT must advance through undivided cortical cells, which should have previously adjusted their physiology and cellular functions, as it has been described in *M. truncatula* and *L. japonicus* nodulation, where the first division events occur in cells located at three to five layers away from the epidermal infection. In contrast, nodulation in *P. vulgaris* is an excellent model to study the straight passage of the IT from the root hair to the subepidermal cortical (for simplicity, here referred as S-E) cell layer, where cells are potentially committed to divide [4,68].

In plant cell cytokinesis initiates at the anaphase of the cell cycle and depends on the de novo formation of a disk-like endomembranous structure, known as the cell plate, constituted by an incipient plasma membrane cross-bounded by cell wall precursors. The onset of the cell plate biogenesis takes place at the center of the plane of cell division by the continuous coalescence of Golgi-derived vesicles, thus creating a disk-shaped tubulovesicular network that will grow by centrifugal expansion towards the periphery of the cell. The cytokinesis process culminates with the fusion of the cell plate membrane with the parental plasma membrane and the separation of the two daughter cells [69]. One of the proteins required for the vesicle fusion events that lead to the cell plate formation is KNOLLE, a plant cytokinesis-specific syntaxin (QaSNARE), which contains one transmembrane domain [70,71]. Transcription of the *KNOLLE* gene occurs at the G2/M transition phase of the cell cycle [72,73], and the protein is initially detected in large cytoplasmic patches in mitotic cells at anaphase. At the early telophase stage, after a vesicular fusion event, KNOLLE remains in the forming cell plate [70]. At the end of cytokinesis, KNOLLE is targeted to the vacuole for degradation [74].

To gain insights into the dynamics of the crossroads between a nascent infection thread and the first cell division event in *P. vulgaris* roots, we designed a microscopic approach that allows for the visualization of the nuclei, the cell walls, the IT progression and the cell cytokinesis status at the infection site. The latter was assessed in transgenic roots expressing the cell-plate specific marker YFP-*Pv*KNOLLE. We have found that the epidermal infection process correlated with the reactivation of the cell cycle in S-E cells underlying the root hair that harbors an IT. As the infection progressed, the S-E cells gradually became shorter. During the formation of a microcolony in a curled root hair and the IT elongation through the body of the root hair, the nuclei in the S-E cells were mainly at the center of the cell, indicative of preparation for mitosis (G2 phase). As the IT extends towards the base of the root hair, YFP-*Pv*KNOLLE led us to visualize a nascent cell plate in an adjacent S-E cell, indicating it was in early telophase. We also observed that the tip of the IT was at the base of the root hair at the time the underlying S-E cell presented a cell plate that apparently had concluded its expansion, suggesting a late cytokinesis stage, whereas it seemed that the IT penetrated the cell when cytokinesis was concluding.

## 2. Results

### 2.1. Epidermal Infection Progression Can Be Described in Four Stages (I–IV)

**Figure 1:**
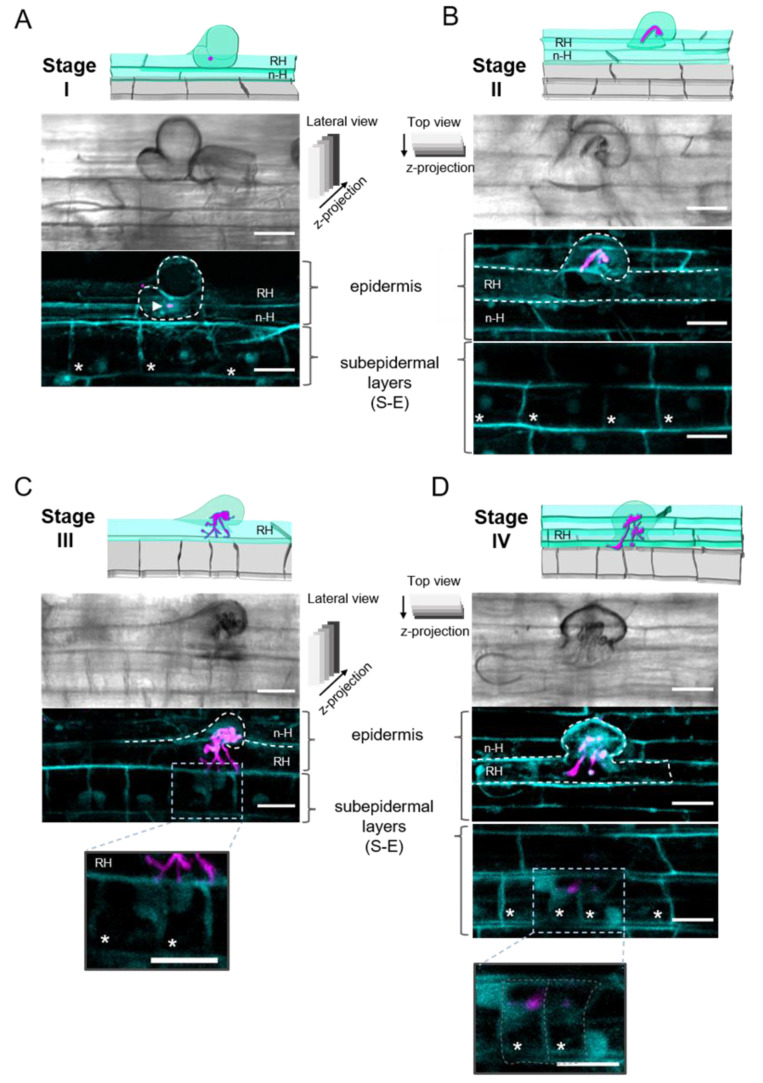
The four stages involved in rhizobia infection in *Phaseolus vulgaris* roots. Schematic representation and 2D projections of images from the epidermal infection sites (stages I to IV) in *P. vulgaris* wild-type roots inoculated with *R. etli* CE3-DsRed pMP604 and harvested at 4 days post-inoculation (dpi). Corresponding differential interference contrast (DIC) images are shown in upper panels of each set of 2D images. Images are representative of each infection thread (IT) progression stage. (**A**) Stage I. Formation of the microcolony in a curled root hair. The arrowhead points to the clumps of rhizobia (magenta) trapped in the fold of a curling root hair. (**B**) Stage II. IT that harbors rhizobia. The IT elongates in an anticlinal orientation, towards the base of the root hair. (**C**) Stage III. The tip of the IT reaches the base of the root hair, but it does not exit the cell. Inset: a closer view of a section in the image in C; it shows the boundary cell wall that separates the base of the root hair from the neighboring subepidermal (S-E) cells. The tips of three IT branches are next to the autofluorescent cell walls, but no rhizobium-derived fluorescent signal is observed in S-E cells, indicating that the IT has not penetrated. (**D**) Stage IV. A root hair harboring a branched IT is observed. Two of the IT branches have penetrated the S-E underlaying cells, respectively. Inset: a closer view of a section in the image in (**D**); 2D projections of images were captured from (**A**) and (**B**) from a lateral view (i.e., z-stacks of a curled root hair and the S-E cells, captured from a longitudinal perspective) and (**B**) and (**D**) from a top view (i.e., z-stacks collected from the top of a curled root hair to the S-E layer), as indicated. The 2D projections of stacks from a top view are presented in two groups, corresponding to the epidermis and the subepidermal cortical layer, respectively. The contour of the cells was detected by cell wall autofluorescence (blue). *R. etli* CE3-DsRed pMP604 is in pseudo-color magenta. Dashed white lines indicate the contour of curled root hairs, and S-E cells in D. RH = root hair, n-H = non-hair epidermal cell. * Indicate the S-E cell layer underlying the root hair harboring an IT. Bars = 20 µm.

For a better appreciation of the IT progression, we used a rhizobial strain expressing the fluorescent protein DsRed [75]. To visualize the cells at the epidermal infection site, we took advantage of the cell wall autofluorescence [76,77], as illustrated in 2D projections of confocal microscopy stacks of images (Figure 1).

For practicality, we divided the infection progression in four stages. Stage I corresponded to the formation of a microcolony, observed as clumps of rhizobia in the fold of a curling root hair, thus creating an infection chamber (Figure 1A). Location of the microcolony was confirmed, as illustrated in Supplementary Figure S1A. The curling zone of the root hair is laying forward on the neighboring non-hair epidermal cell, and the S-E cells are the underlying cells, as was resolved in an orthogonal 3D projection (Supplementary Figure S1B,C). Stage II involved a growing IT, which elongated in an anticlinal orientation, towards the base of the root hair, and eventually branched (Figure 1B). In Figure 1C, the IT is branches and reaches the base of the root hair, but none of the IT branches have exited the root hair, as confirmed by an analysis of orthogonal 3D projections (Supplementary Figure S2); therefore, we assigned it as an IT progression stage III. As illustrated in Figure 1D, we defined an IT at stage IV when it has penetrated an S-E cell.

### 2.2. Subepidermal Cells (S-E) Underneath the Infection Site Become Shorter as the Infection Thread (IT) Progresses

**Figure 2:**
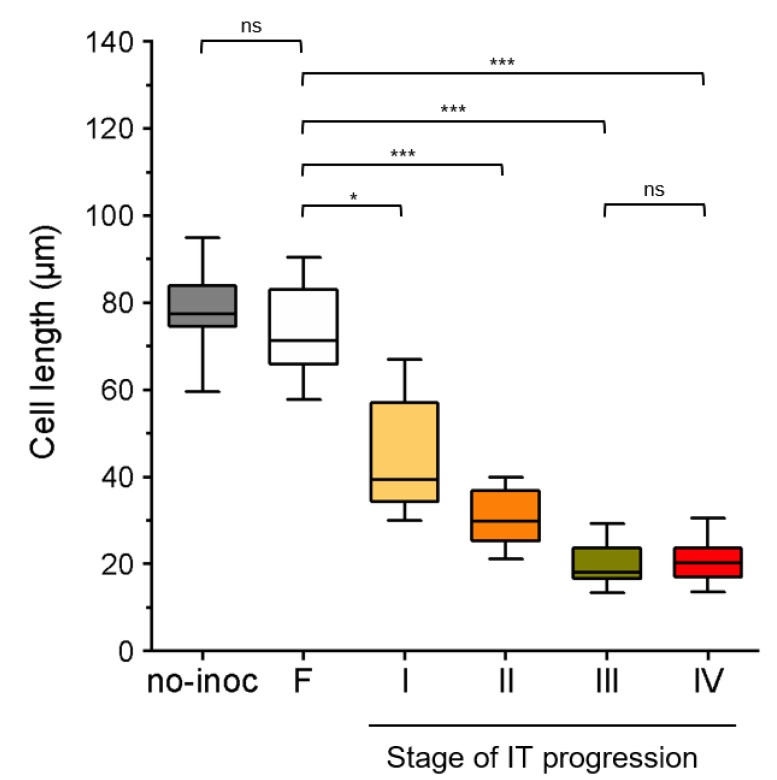
Length of subepidermal (S-E) cells in uninoculated and rhizobium-inoculated *P. vulgaris* roots at stages I–IV of IT progression in epidermal infection sites. Boxplot graph of cell length determined in series of independent 2D projections of S-E cells from the initial differentiation zone (susceptible for rhizobia infection) of uninoculated (no-inoc) roots or from S-E cells flanking (F) or underneath an epidermal infection at stages I–IV of the IT progression. Average (± standard deviation of the mean, SD) of length values: no-inoc, 78.5 ± 9.7, *n* = 14 cells; F, 73.2 ± 9.6, *n* = 23 cells; stage I, 46.5 ± 12.6, *n* = 23 cells; stage II, 30.9 ± 6.5, *n* = 19 cells; stage III, 20.2 ± 4.6, *n* =14 cells; 20.5 ± 4.8, *n* = 18 cells. Data were compared using a Kruskal–Wallis test, and Dunn’s multiple comparison test was performed as a post hoc analysis considering the length of flanking (F) S-E cells as a control. The Mann–Whitney test was used for comparing data from no-inoc and F samples, or data from samples at stage III and IV, which show there is no significant difference, respectively. In graphs, * and *** indicate statistically different with *p* < 0.05 and *p* < 0.0001, respectively; ns, no significant difference (*p* > 0.05).

In a detailed analysis of representative images of the epidermal infection stages, we observed that at stages I and II, the S-E cells seemed to be shorter than those not participating in an epidermal infection. To provide insights into such observation, we performed a comparative analysis of length measurements obtained from S-E cells underlying an epidermal infection site and those of S-E cells flanking the site. As a reference, we also measured S-E cells located at the differentiation zone, susceptible to rhizobia infection, in uninoculated roots; no significant difference was found with respect to S-E cells flanking the infection site (Figure 2). In roots inoculated with rhizobia, S-E cells underneath an epidermal infection exhibited a notorious tendency to become smaller as the infection progresses from stage I to IV. In comparison to the length of S-E flanking cells, there is a reduction of approximately 42, 60, 74 and 75% in averaged cell length at stages I to IV, respectively (Figure 2).

The S-E cell size reduction during infection suggests that, in *P. vulgaris* nodulation, the turning on of the reactivation of cell cycle program occurs before or during the microcolony formation (stage I). The size of S-E cells at stage II may coincide with the cell conditioning for later steps of cell division, which may take place as the IT progresses to stages III and IV.

### 2.3. The Position of the Nucleus in the Subepidermal Cells Underlying the Epidermal Infection Site Correlates with the Infection Thread (IT) Progression

**Figure 3:**
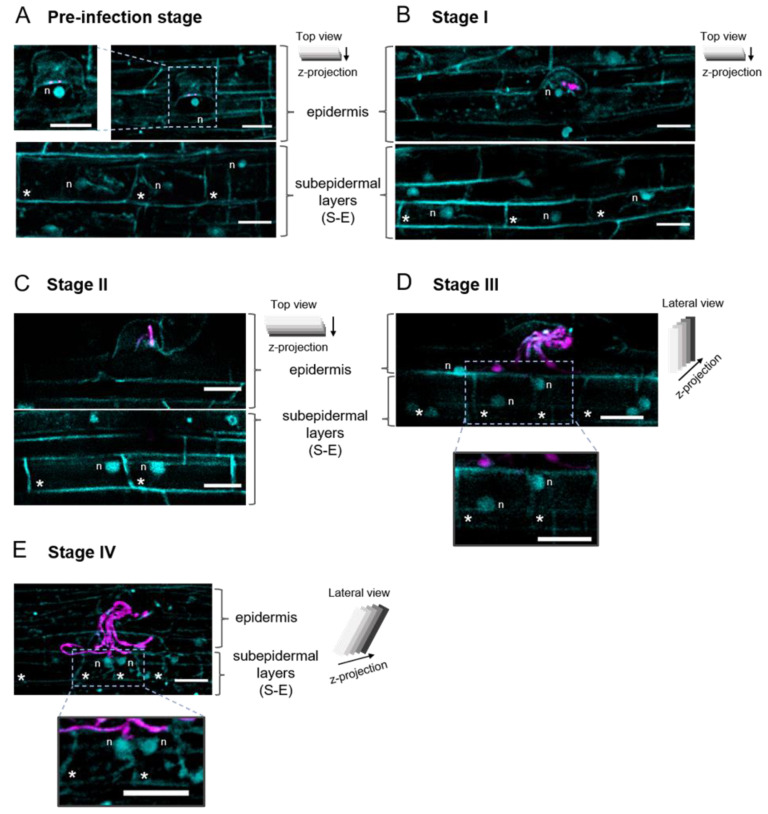
Visualization of the nucleus position in cells at epidermal infection site. The 2D projections of *Phaseolus vulgaris* roots expressing the nuclear marker NLS-mTurquoise2 or stained with DAPI (blue in both cases) and inoculated with *R. etli* CE3-DsRed pMP604 (4 dpi). Images are representative of each infection thread (IT) progression stage. Position of the nucleus is variable but correlates with the infection progression. Root hairs at the (**A**) pre-infection stage, as revealed by the detection of rhizobia (magenta) attached to the apical surface of the root hair apical zone. The nucleus was detected at the apical zone, close to the bacterial attachment spot. (**B**) Stage I. The nucleus moves to the nascent infection site. (**D**) Stage III. As the IT progresses, the nucleus relocates to the base of the root hair. In S-E cells, the nucleus is located at different positions in the cells (see Figure 4). (**B**) Stage I. The nuclei are predominantly located at the center of the cell. (**C**) Stage II. The nuclei are observed at the apical zone of the subepidermal (S-E) cells, although at this stage, they tend to be located at the center (see Figure 4). (**D**,**E**) Stages III and IV, the nuclei are distributed in different locations, with a tendency to be at the apical zone at stage IV. For a better appreciation, insets were added in panels (**A**,**D**,**E**), respectively. Nuclei (blue in all panels) in (**A**,**B**,**E**) are stained with DAPI; nuclei in (**C** and **D**) are labeled with NLS-mTurquoise2. Cell wall autofluorescence was also visualized in blue. *R. etli* CE3-DsRed pMP604 is in pseudo-color magenta. n = nucleus, RH = root hair, n-H = non-epidermal hair. * Indicate the S-E cell layer underlying the root hair harboring an IT. Additional information in panels (**A**–**E**) is as described in the legend of Figure 1. Bars = 20 µm.

According to van Spronsen et al. [4], at initial stages of *P. vulgaris* nodulation, cortical cells that were activated for division contained swollen nuclei that were situated in the center of the cell. To explore the position of the nucleus in S-E cells at the epidermal infection site, we analyzed rhizobium-inoculated *P. vulgaris* wild-type roots stained with DAPI and transgenic roots expressing the construct *p35S*::*NLS-mTurquoise2*, as illustrated in Figure 3. Seeking epidermal infection events, we found root hairs with one or two rhizobia attached to the surface of the root hair apical zone, indicative of a pre-infection stage (Figure 3A). In those cells, the nucleus was at the apical zone, close to the bacterial attachment spot (Supplementary Figure S3A), as previously described [78]. Meanwhile in root hairs at stages I (Figure 3B) and II, the nucleus was moved nearby the nascent infection site or close to the tip of the IT, respectively. At stages III (Figure 3D; Supplementary Figure S3B) and IV, it was located at the base of the root hair, similar to what happens at early stages of rhizobial infection in *M. truncatula* and *L. japonicus* [38,78,79].

**Figure 4:**
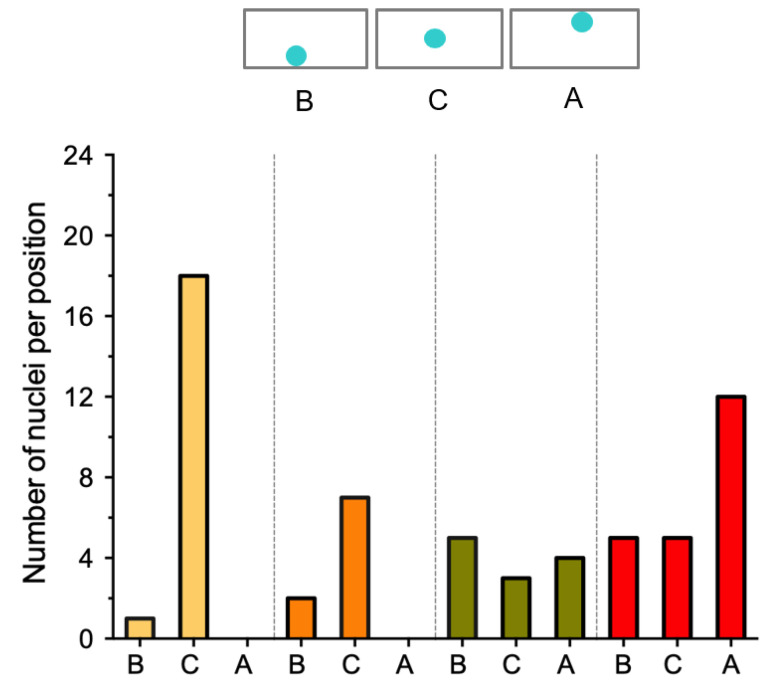
Distribution of the nuclei position in subepidermal (S-E) cells at stages I–IV of the IT progression. Graphic representation of the nuclei position in cells at each of the IT progression stages. Nuclei were either stained with DAPI or expressing NLS-mTurquoise2. Images were captured from 17 independent roots inoculated with *R. etli* CE3-DsRed pMP604. Number of cells (*n*) analyzed: stage I, *n* = 19; stage II, *n* = 9; stage III, *n* = 12; and stage IV, *n* = 22. Nuclei from each stage were classified in three groups, depending on their position in the cell: B, basal; C, central and A, apical.

In S-E cells underlying an epidermal infection, the position of the nucleus was variable, although it correlated with the infection stage (Figure 3 and Figure 4). At stages I and II, the nuclei were mainly at the center of the cell (Figure 3B,C and Figure 4). At stages III and IV, the nuclei were observed distributed at the basal, central, and apical sides of the cells (Figure 3D,E and Figure 4), although at stage IV they were predominantly (50%) located at the apical side of the S-E cells (Figure 4). Interestingly, in those S-E cells invaded by the IT, the nucleus was frequently found nearby the penetration site, which may be related to a cell reorganization in preparation for the IT penetration.

### 2.4. Cell Plate-Labeling with YFP-PvKNOLLE Indicates That the Infection Thread (IT) Penetrates the Subepidermal Cell (S-E) at Late Cytokinesis

We further explored whether the IT penetration to the subepidermal layer occurs before the target cell has concluded the division process by tracing the cytokinesis marker KNOLLE. We first identified the *P. vulgaris KNOLLE* gene (Phvul.004G077900) and proceeded to analyze the promoter activity of a fragment of 1.76 kb upstream the start codon (p*PvKNOLLE*), which includes three Mitosis Specific Activator (MSA [80]) elements at 50 bp upstream the transcription initiation site. We confirmed that p*PvKNOLLE* activity was limited to the root apical meristem and events of lateral root formation, as well as in the development of nodule primordia and young nodules (Supplementary Figure S4A–E). Of particular interest for our study, p*PvKNOLLE* activity highlighted the dividing S-E cells underlying an epidermal infection (Supplementary Figure S4F). Hence, p*Pv* *KNOLLE* confers a specific spatio-temporal activity in dividing cells. We therefore proceeded to analyze *P. vulgaris* transgenic roots bearing the construct p*PvKNOLLE*::*YFP-PvKNOLLE*. Cytokinetic cells were easily detected in the root apical meristem (RAM) through visualizing YFP-*Pv*KNOLLE, which pinpoints the cell plate in formation and provides information regarding the plane of cell division. As expected, abundant cells with anticlinal division were observed, but very few had periclinal orientation (Supplementary Figure S5A,B). According to their location, the latter may correspond to division of cortex initial cells (Supplementary Figure S5A). Oblique/flanking periclinal divisions [81] were rarely observed. Additional information can also be depicted from visualizing YFP-*Pv*KNOLLE, such as the distinguishing transition and elongation zones from the RAM (Supplementary Figure S5A), measuring the distance separating the cell plates in contiguous cells (Supplementary Figure S5C) and monitoring mitotic cells from telophase until the cytokinesis is completed (Supplementary Figure S5D). Last, a comparison in the length and width of cells in the RAM *versus* those in the transition zone can be done (Supplementary Figure S5E). We also benefited from the restricted expression of YFP-*Pv*KNOLLE in cytokinetic cells to track the cell plate expansion in S-E cells at the epidermal infection site.

**Figure 5:**
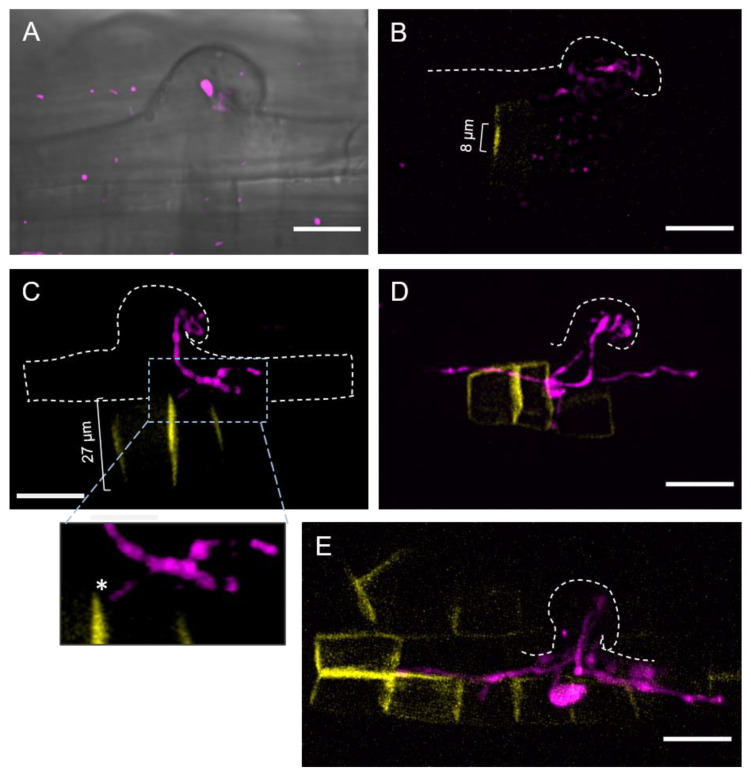
Visualization of the cell plate in subepidermal (S-E) cells underlying the epidermal infection at stages II and IV of the infection thread (IT) progression. The 2D projections of the epidermal infection site in *P. vulgaris* transgenic roots expressing YFP-*Pv*KNOLLE and inoculated with *R. etli* CE3-DsRed pMP604 (4 dpi). Images are representative of each IT progression stage. (**A**) Stage I. Fluorescence image was merged with the differential interference contrast (DIC) image to highlight the position of the microcolony in the context of the curled root hair. No YFP-*Pv*KNOLLE signal was detected in the underlying S-E cells, indicating they are not cytokinetic. (**B**) Stage II. YFP-*Pv*KNOLLE is labeling a nascent cell plate, suggesting the S-E cell is at early telophase. (**C**) Stage III. The cell plate is expanded to the periphery of the S-E cell underlying the infected root hair. The tip of an IT branch has reached the base of the root hair and it is close to the zone of the putative fusion of the cell plate with the parental plasma membrane, indicated with an asterisk in the inset. (**D**) Stage IV. The IT has exited the root hair to penetrate a post-cytokinetic underlying S-E cell, meaning the cell plate formation has concluded. (**E**) The IT invades several cytokinetic cells adjacent to the epidermal infection site. Length of the respective cell plate in (**B**,**C**) is indicated. *R. etli* CE3-DsRed pMP604 is in pseudo-color magenta. Dashed white lines indicate the contour of curled root hairs. Bars = 20 µm.

At the microcolony-forming stage I, no YFP-*Pv*KNOLLE signal was detected (Figure 5A), implying that neighboring S-E cells were not cytokinetic. The images presented in Figure 5B,C correspond to epidermal infections at stage II and III of the IT progression, respectively. The infected root hair at stage II of the IT progression was lying forward on the epidermis and the tip of the IT branches were close to the base of the root hair, as can be seen in Figure 5B and Figure S6A. A nascent cell plate (8 µm length) labelled with YFP-*Pv*KNOLLE was observed in the S-E cell adjacent to a root hair that housed a branched IT (Figure 5B and Supplementary Figure S6A). According to van Oostende-Triplet et al. [82], the de novo formed disk-shaped tubulovesicular structure is typically 5.5 µm in length. It then expands rapidly, reaching 15 µm in length to further slow down until the cell plate formation is completed at late telophase. At this point, the cell plate is 20–35 µm in length. In that regard, our detection of a cell plate of 8 µm in length indicated that the S-E cell at infection stage II was cytokinetic at the early telophase. In the image illustrating an epidermal infection at stage III (Figure 5C), the tip of one of the IT branches is located close by the basal membrane of root hair, and its position leads to predict the putative penetration site (Figure 5C and Figure S6B). In the to-be invaded S-E cell, the length of the cell plate appeared to be 27 µm, suggesting a late cytokinesis status. Figure 5D illustrates a late stage IV of IT progression. Here, YFP-*Pv*KNOLLE is labeling a post-cytokinesis cell plate structure, whose lumen seems wider (Supplementary Figure S6C) and may be in transition to become the apoplast that will separate the daughter cells. Figure 5D also shows the passage of a branch of the IT from one side of the former post-cytokinetic cell plate structure to further elongate through the contiguous daughter cell. Orthogonal 3D projections offered a better perspective of the crossing event (Supplementary Figure S6C). Last, an image of a later stage of the IT progression is presented (Figure 5E), showing how branches of an IT can penetrate several cells forming a young nodule primordium.

Taken together, our results provide strong evidence showing that, in *P. vulgaris* nodulation, the IT does not penetrate undivided cortical cells, but rather those that have reactivated their cell cycle and are concluding cytokinesis.

## 3. Discussion

To gain a better understanding of the crossroads between infection and organogenesis at early stages of nodulation in *P. vulgaris* roots, we undertook a microscopy approach to document the passage of the IT from the root hair to the underlying subepidermal cortical cell layer (S-E), where the first CCD occurs. This biological circumstance is unique. In the nodulation models *L. japonicus* and *M. truncatula*, those events are separated by two to five cortical layers, respectively [3,4].

**Figure 6:**
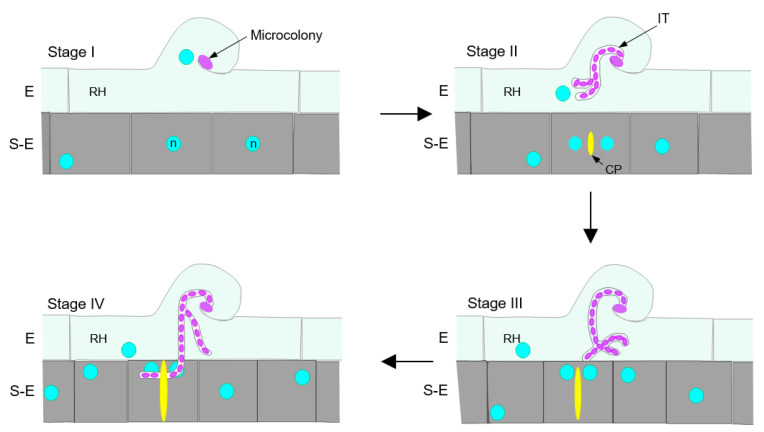
Schematic representation of the dynamics between the infection thread (IT) progression and the reactivation of the cell cycle. Infection thread (IT) progression was divided in stages I–IV, in which cells become successively shorter. Stage I: Rhizobia (magenta) is forming a microcolony, and the cell nucleus (circles in blue) is mainly at the center of the cell. No cell plate (CP, in yellow) is distinguished. Stage II: IT is formed and branched. A nascent CP is observed when the IT tip is close in the body of the RH. Stage III: the tip of the IT reaches the base of the RH and the CP use to be at the end of expansion process, suggesting a late cytokinesis in expansion. Stage IV: the IT has penetrated an adjacent subepidermal (S-E) cell and may cross the CP that is fully expanded, indicative of a post-cytokinetic status. E, epidermis.

Regarding the IT progression, even though it is difficult to visualize it through a coarse root hair, as in *P. vulgaris*, we found the process is quite similar to the IT development in *L. japonicus* and *M. truncatula* [6,83]. Autofluorescence around the microcolony (Figure 1A and Figure 6) allowed us to predict an infection chamber. According to Fournier et al. [32], the infection chamber provides the environment for the initial proliferation and conditioning of rhizobia for their entry to the root hair and favors the cell wall remodeling and plasma membrane invagination that give raise to the initial IT structure [32]. In *M. truncatula*, it takes approximately 15 to 18 h to initiate an IT from a curled root hair [32]. Whilst the microcolony is forming, the nucleus moves towards the tip of the root hair, to subsequently move through the body of the root hair, presumably guiding the IT towards the base of the cell (Figure 3 and Figure 6) [78,79,84].

As the IT progresses, the cortical cells adjacent to the infection site must be committed and be prepared for the IT invasion [10,85]. The fate of the IT exiting the root hair in *P. vulgaris* is to penetrate a S-E dividing cell, whereas in *M. truncatula*, *M. sativa*, *L. japonicus* and other nodulation models, the IT faces S-E cells that have reactivated their cell cycle but do not enter mitosis [63]. This reactivation is indicated by the induced formation of a cytoplasmic bridge in the outermost cortical layers opposite to an epidermal infection site [4,85,86]. In highly vacuolated cells, such as root cortical cells and tobacco BY-2 cells, the cytoplasmic bridges or phragmosome are related to the cell division [87]. In this type of cell at the G1 phase, the vacuolar system is fragmented. At the G2 phase, the nucleus is displaced to the central region of the cell and a sheet of cytoplasm and arrays of microtubules are radiating from the nucleus to the cell periphery. Afterwards, a transvacuolar cytoplasmic disc gradually accumulates across the central region of the cell, forming a cytoplasmic bridge, where later the phragmoplast will form [87,88,89]. In the nodulation of Medicago, *Vicia sativa, Pisum sativum* and *L. japonicus*, the cytoplasmic bridge, also designated as pre-infection thread, provides the path to cross the root cortex until reaching the forming nodule primordium [4,85,86]. Moreover, it has been postulated that formation of the pre-infection thread is involved in the weakening and deformation of the cell wall at the site where the IT will gain access to the neighboring cell [86]. No pre-infection threads have been observed in *P. vulgaris* roots, by means of histological examination [4]. Though it would be interesting to further address that issue by studying the vacuolar and microtubule dynamics in S-E cells upon inoculation with rhizobia, which is a matter of future research. However, we gained some insights into that direction by analyzing the cell length and nucleus distribution in *P. vulgaris* S-E cells underlying an epidermal infection. At stage I of infection, when a microcolony was observed in a curled root hair, these S-E cells were shorter than flanking cortical cells, and their nuclei were mostly at the center of the cell (Figure 2, Figure 4 and Figure 6). These parameters suggest the cell cycle was reactivated in those cells and potentially progressed to the G2 phase [90], though the appropriate analysis needs to be performed to better estimate the cell cycle reactivation dynamics and duration. It is noteworthy to mention that similar criteria were applied to distinguish dividing from undividing cells in *M. truncatula* roots overexpressing *enod40,* where an extensive inner cortical cell division was observed in the absence of rhizobia [76]. Moreover, the description of cortical cell division deficiency and the rhizobium-independent CCD phenotypes of a series of mutants in gene-silencing experiments often refers to the detection of a row of cortical cells shorter than in the control roots [19,60,67,91]. The same experimental strategy was used to demonstrate the mitogenic activity of purified Nod factors and the effect of plant hormones on CCD in nodulation [92,93,94]. In that regard, it would be interesting to have an estimation on the delay between the cell cycle reactivation and the conclusion of cytokinesis in those cortical cells participating in the nodulation process. Even though it certainly will be a difficult task in roots as thick as those in *P. vulgaris*, such an analysis would set the experimental conditions to address the molecular and cellular mechanisms underlying the cell cycle reactivation. As a reference, the duration of S/G2 and M phases within the root apical meristem and the transition zone in Arabidopsis roots was approximately 5 h, where mitosis had an estimated duration of 20–25 min [95].

Our conclusions on the cell cycle activation of S-E cells were further supported by the analysis of the epidermal infection in transgenic roots expressing the cytokinesis marker YFP-*Pv*KNOLLE, in which formation and expansion of the cell plate was easily traced. We found that, as the IT progressed towards the base of the root hair (stage II), a cell plate became visible in the underlying S-E cell (Figure 5 and Figure 6). In tobacco BY2 cells, a nascent cell plate was detected at early telophase, as a disk-shaped structure of approximately 5 µm in length [82]. Detection of a YFP-*Pv*KNOLLE-labelled structure with a similar size in S-E cells underlying an epidermal infection stage II led us to conclude that those cells were at telophase.

Visualization of the IT passage from the base of the root hair to the neighboring S-E cell was the main focus of our work. With that aim, we documented the cell plate expansion during late telophase and cytokinesis, finding a correlation between the position of the IT tip at the base of the root hair (IT progression stage III) at the time the underlying S-E cell presented a cell plate that apparently had concluded its expansion, suggesting a late cytokinesis (Figure 5C, Figure 6 and Figure S6B). At a later stage of the IT progression (stage IV, as illustrated in Figure 5D and Figure S6C), the IT had already penetrated the underlying S-E cell, when the cell was at a post-cytokinetic status. The IT was further expanded to the next S-E cells (Figure 5D,E and Figure 6).

In summary, we presented a punctual description of the crossroad between the IT pre- and post-penetration of the neighbor cell and the cytokinesis progression in the targeting cell (Figure 6). Our findings provide strong evidence supporting the notion of a coordination between the IT exit from the root hair and the progression of cell cycle in the neighboring cell that will give access to the IT. We also demonstrated that, in *P. vulgaris* roots, the IT is capable of penetrating a cytokinetic cell. In that regard, it is reasonable to postulate that the signaling induced at early stages of *P. vulgaris* nodulation leads to a direct molecular instruction not only intended to reactivate the cell cycle in S-E cells, but to proceed to cell division, a challenging question that may be worth exploring in future studies.

## 4. Materials and Methods

### 4.1. Plants and Bacteria Growth Conditions

*Phaseolus vulgaris* cv. Negro Jamapa (common bean) seeds were obtained from local farmers in Morelos, Mexico. Seeds were surface sterilized with 70% alcohol (1 min) and 20% commercial chlorine (5 min). Subsequently, seeds were germinated at 28 °C for 48 h in the dark, as previously described [75]. Two days post-germination (dpg) seedlings or composite plants were transferred to pots containing vermiculite and inoculated with *Rhizobium etli* CE3-DsRed pMP604 [75], diluted in 10 mM MgSO4 to an OD600nm of 0.05. Plants were grown under controlled environment conditions (28 °C, 16 h/8 h photoperiod), were watered with nitrogen-free Fåhraeus medium [96] and harvested at the indicated time points. Composite plants with transgenic roots were generated using the *Agrobacterium rhizogenes* K599-dependent transformation protocol, as previously described [25]. *A. rhizogenes* and *R. etli* strains were grown at 30 °C for 48 h in LB or PY media, respectively.

### 4.2. Plasmid Constructions

The sequence of *P. vulgaris* KNOLLE gene was identified in the Phytozome database (https://phytozome-next.jgi.doe.gov/ (accessed on 30 June 2018), accession number Phvul.004G077900). For the analysis of *PvKNOLLE* promoter (p*PvKNOLLE*) activity, the sequence of a 1.76 kb fragment upstream from the start codon was amplified by PCR from genomic DNA using gene-specific primers. The PCR product was then cloned by recombination (Gateway^®^ LR Clonase™ II Enzyme Mix, Invitrogen, Waltham, MA, USA) into the plant vector pBGWFS7 to generate the p *PvKNOLLE*::*GFP-GUS* transcriptional fusion. To construct the expression cassette p*PvKNOLLE*::*YFP*-*PvKNOLLE*, the *PvKNOLLE* coding sequence (953 bp) was amplified in a RT-PCR reaction using total RNA from *P. vulgaris* root apex and the primers PvKNOLLE-ATG and PvKNOLLE-stop. The PCR product was then recombined into the plant vector pEarleyGate 104 to create the cassette *p35S::YFP-PvKNOLLE*. The last step consisted in the substitution of p35S sequence with the p*PvKNOLLE* fragment using EcoRI and NcoI and ligation strategy, leading to plasmid, pEarleyGate104_ p*PvKNOLLE*::*YFP*-*PvKNOLLE*. To generate the expression cassette p35S::NLS-mTurquoise2, a chimeric cDNA coding for NLS-mTurquoise2 (751pb) was PCR amplified using the primer NLS-Turquoise UP, which comprises 24 nucleotides coding for the NLS sequence in frame with 18 nucleotides of the 5′ end of the mTurquoise2 coding sequence, and the primer Turquoise LW. Plasmid pmTurquoise2-ER was used as template. NLS-mTurquoise2 cDNA was subcloned by recombination into the vector pK2GW7 to generate the transcriptional fusion p*35S::NLS-mTurquoise2* (*pK2GW7-NLS-mTurquoise2*). All cloning steps were performed in *E. coli* DH5 α, confirmed by sequencing and electroporated into *A. rhizogenes* K599. The primer information is listed in Supplementary Table S1.

### 4.3. Fixation and DAPI Staining Conditions

*P. vulgaris* wild-type roots were harvested at 4 days post-inoculation (dpi) with *R. etli* CE3-DsRed pMP604 and were fixed overnight in 4% paraformaldehyde (PFA) in 80 mM PIPES pH7 solution. Then, samples were rinsed three times for 5 min in 80 mM PIPES pH 7.0 [77]. Nuclei in fixed roots were stained using 4′,6-diamidino-2-phenylindole (DAPI, Sigma-Aldrich) at 1 μg/mL in 0.1% (*v*/*v*) Triton X-100 for 30 min in the dark at room temperature and washed three times with water.

### 4.4. Confocal Imaging

*P. vulgaris* wild-type and composite plants were harvested and placed in a modified polystyrene square Petri dish, in which part of the bottom was manually replaced by a glass cover slip (48 × 65 mm). The roots were whole mounted on the coverslip containing 1 ml of Fåhraeus medium, covered with a sweet cellophane sheet and observed in an inverted confocal laser scanning microscope FV1000 (with a 40×/NA 0.75 dry objective). DAPI, mTurquoise2 and autofluorescence of cell wall components were excited with 405 nm and detected at 430–470 nm. YFP and DsRed were visualized with 488 nm and 543 nm excitation, respectively, with fluorescence emission at 505–525 nm and 560–660 nm, respectively. Z-stacks were collected using a resolution of 800 × 800 pixels and were taken at a step size of 0.8–1.0 µm. YFP-KNOLLE images were captured using a 3I Marianas Confocal Spinning Disk Microscope coupled to a Zeiss Observer Z.1 Inverted type (water ×40 objective 0.75 N.A., ZEISS, Jena, Germany).

### 4.5. Image Processing and 3D Visualization

The 2D analysis of z-stack confocal images was carried out with ImageJ/Fiji software (National Institutes of Health, Bethesda, MD, USA). The z-stacks were analyzed using the maximum intensity projection to generate 2D images.

Deconvolution was applied to each stack of images for each channel. The deconvolution was performed using the “Iterative Deconvolve 3D” plugin in ImageJ and “Diffraction PSF 3D” for the creation of theoretical PSF, with 4–10 maximum iterations. To generate the 3D reconstructions, the two-channel z-stack (DAPI or NLS-mTurquoise2 + DsRed; YFP-*Pv*KNOLLE + DsRed channels) was merged and converted to RGB format, using the menu command Image›Type›RGB color. Three-dimensional reconstructions were created using Vaa3D^®^ software (3D Visualization-Assisted Analysis) [97].

### 4.6. Statistical Analysis

To validate the quantitative analysis and confirm the reproducibility of the results, statistical analysis of the data was performed using GraphPad Prism version 6 and considering a series of biological replicates, as indicated. The difference between values from incubation times in time-course experiments were evaluated using Kruskal–Wallis test, Mann–Whitney test, Dunn’s multiple comparison test or Student’s *t-*test, as indicated. *p* values are indicated in the figure legends.

## Data Availability

Not applicable.

## Supplementary Materials

The following supporting information can be downloaded at: https://www.mdpi.com/article/10.3390/ijms23095267/s1.
