# Supplementary material for: Visualization of the Crossroads between a Nascent Infection Thread and the First Cell Division Event in Phaseolus vulgaris Nodulation
Source: Int J Mol Sci. 2022 May 9;23(9):5267. doi: 10.3390/ijms23095267 (PMC9105610; doi:10.3390/ijms23095267)
Supplement: Supplementary file 1 [file ijms-23-05267-s001.zip › ijms-1672644-supplementary.pdf]

## Supplementary Materials

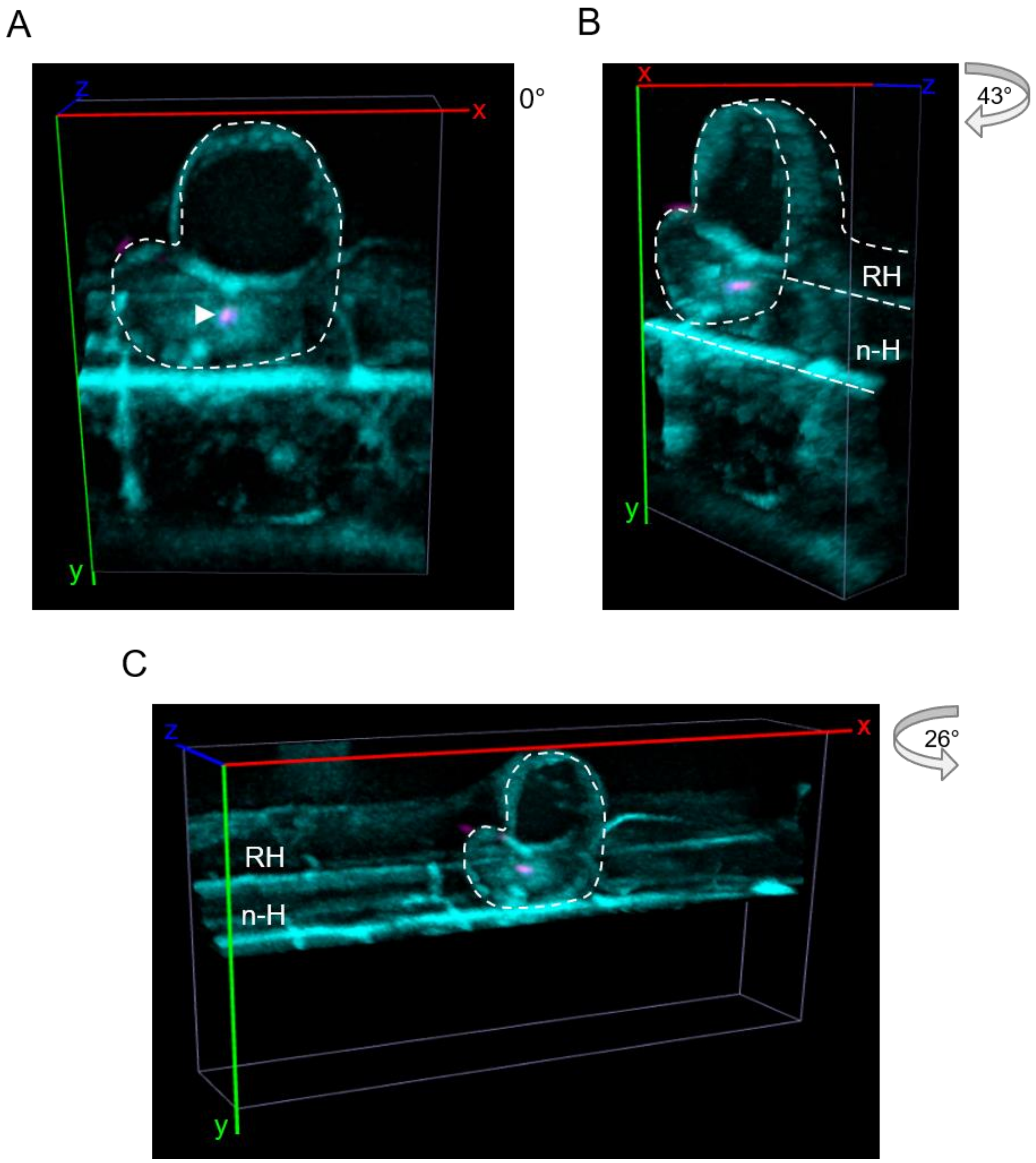


**Supplementary Figure S1.** 3D reconstruction of the epidermal infection site at stage I illustrated in Figure 1A. Reconstruction was created using Vaa3D® software (3D Visualization-Assisted Analysis). The rotation angles are indicated in each panel. The image was reconstructed from z-stacks = 25.2 µm, with a step size = 0.9 µm per slice. RH = root hair, n-H = non-hair epidermal cell.

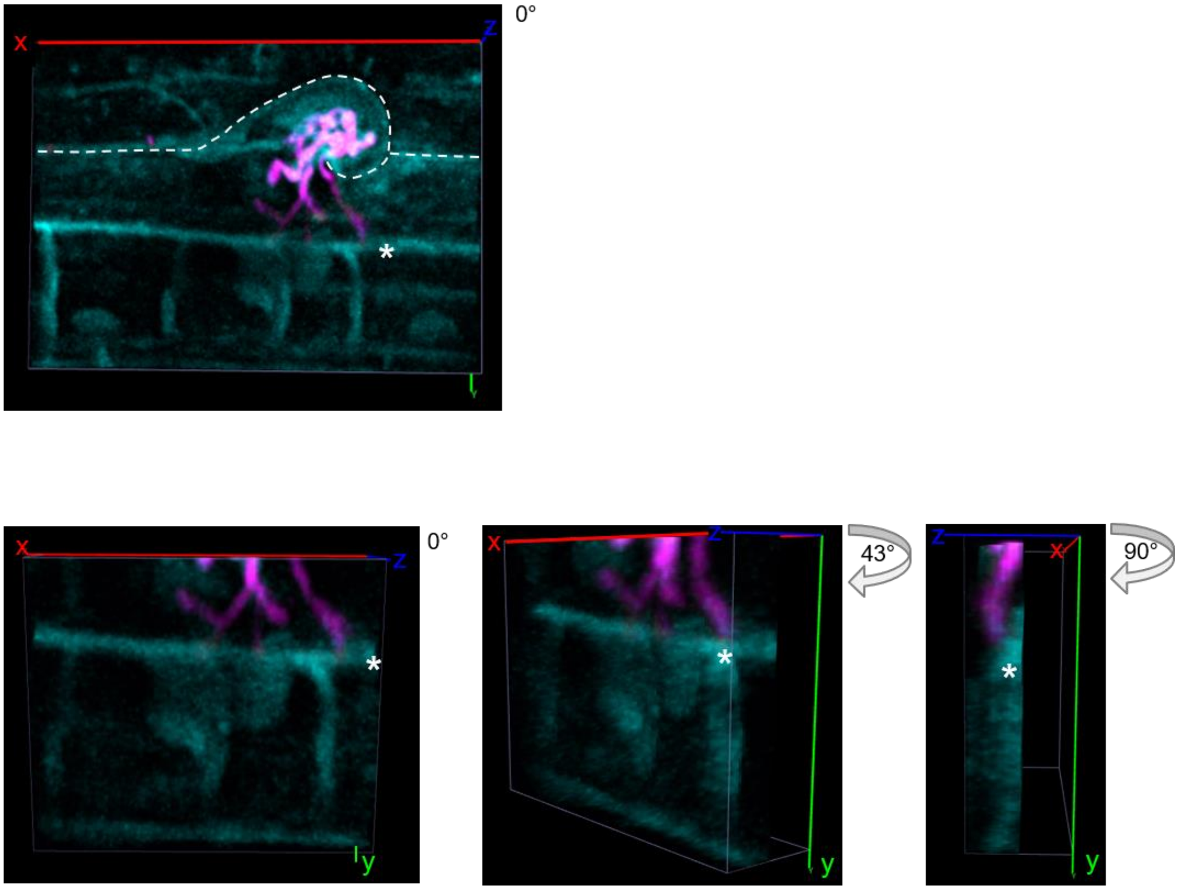


**Supplementary Figure S2.** 3D reconstruction of the epidermal infection site at stage III illustrated in Figure 1C. Reconstruction was created using Vaa3D® software (3D Visualization-Assisted Analysis). The rotation angles are indicated in each panel. The image was reconstructed from z-stacks = 9.6 μm, with a step size = 0.8 μm per slice. * Indicates the boundary cell wall that separates the base of the root hair from the neighbouring S-E cells.

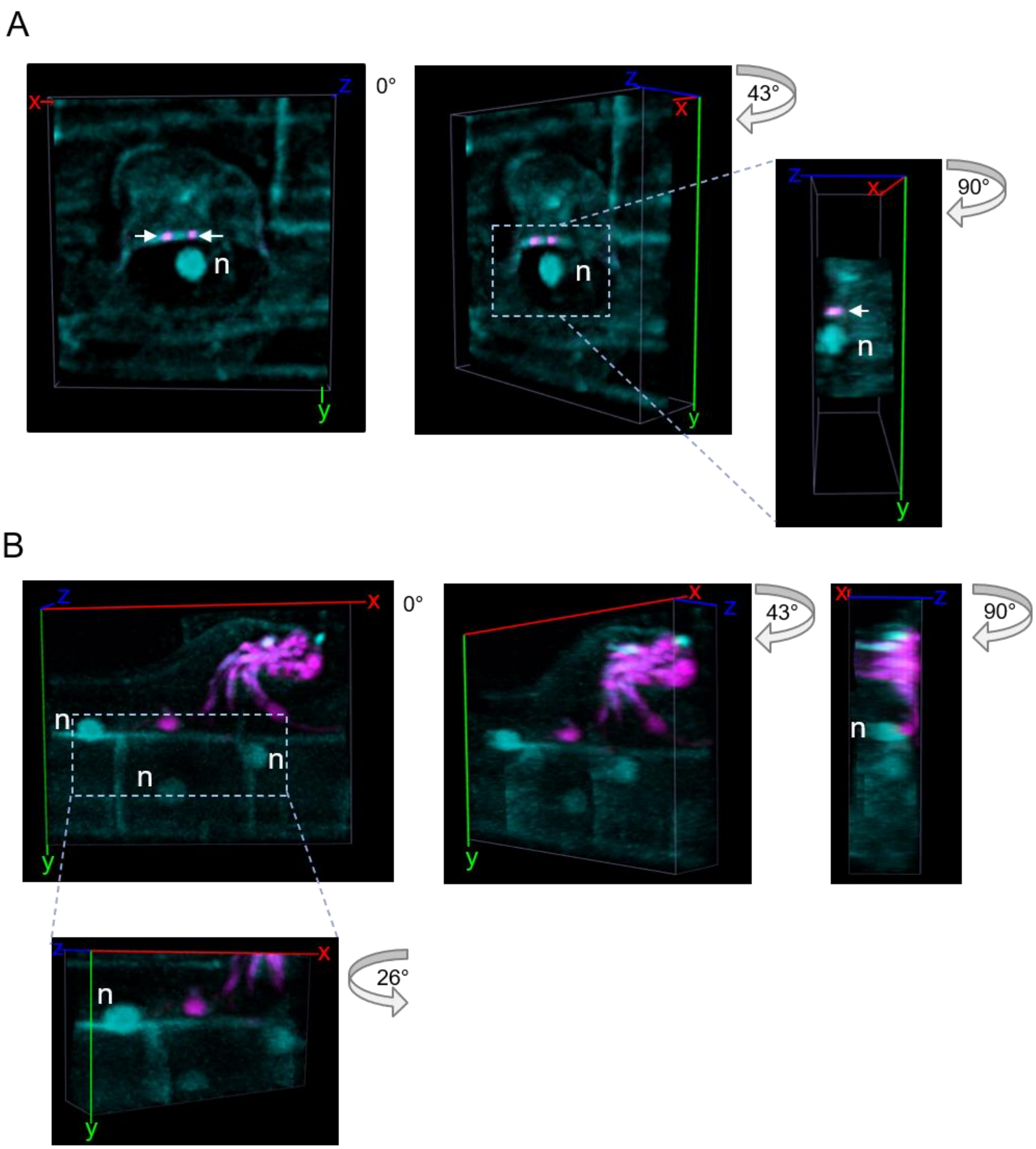


**Supplementary Figure S3.** 3D reconstruction of the epidermal infection site illustrated in Figure 3A and D. Reconstruction was created using Vaa3D® software (3D Visualization-Assisted Analysis). (**A**) Pre-infection stage reconstructed from z-stacks = 6.3 μm, with a step size = 0.8 μm per slice. (**B**) Stage III, reconstructed from z-stacks = 11.6 μm, with a step size = 0.83 μm per slice. The rotation angles are indicated in each panel. n = nucleus. Arrows point to rhizobia attached to the surface of the root hair apical zone.

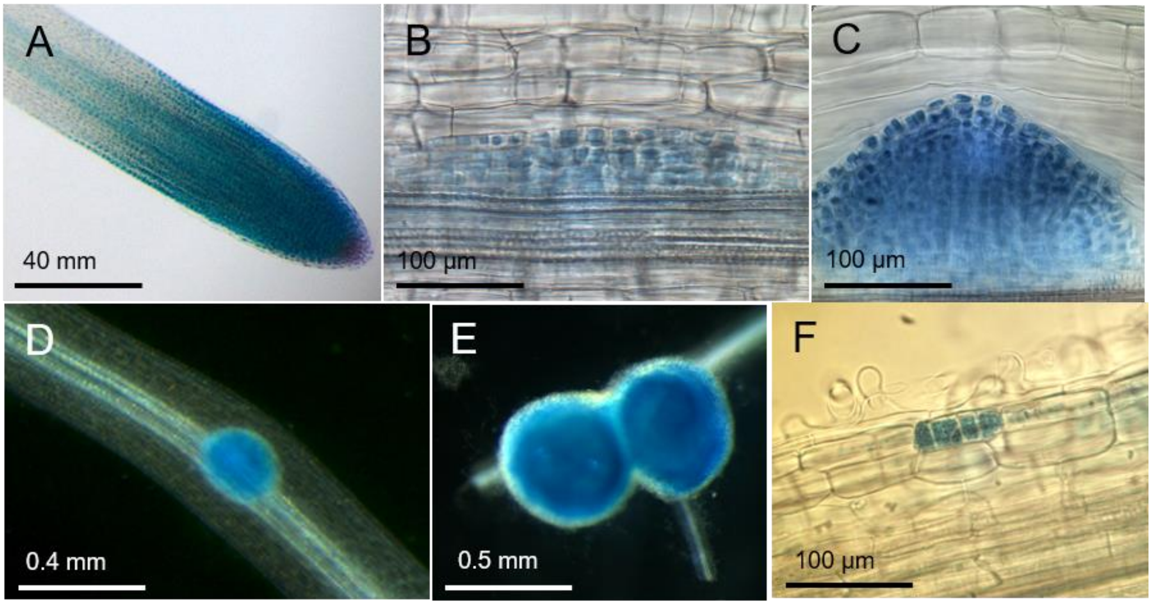


**Supplementary Figure S4.** Spatio-temporal activity of *Pv*KNOLLE promoter in *P. vulgaris* transgenic roots and in nodulation. Promoter activity was detected specifically in meristematic tissues. In the root: (**A**) Root apical meristem (RAM). (**B**) Lateral root initiation. (**C**) Lateral root primordium. In the nodulation process: (**D**) Nodule primordium. (**E**) Young nodule. (**F**) Cortical cell division at the subepidermal cell layer. Promoter activity was determined as histochemical staining of GUS activity.

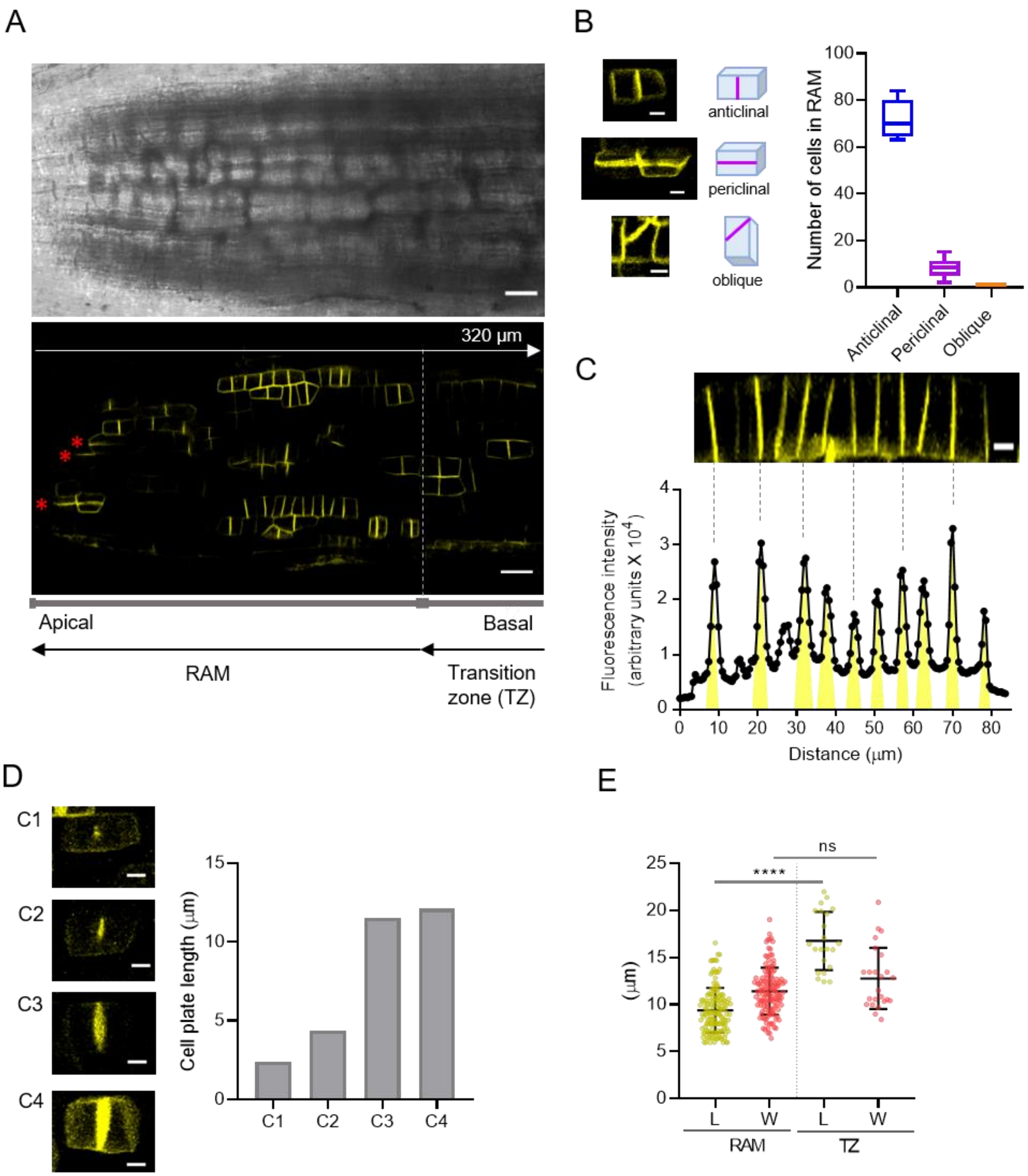


**Supplementary Figure S5.** Brief analysis of the RAM and transition zone in transgenic *P. vulgaris* roots expressing the cytokinesis marker YFP-*Pv*KNOLLE. (**A**) Representative image showing the citolocalization of YFP-*Pv*KNOLLE in the cell plate of dividing cells in the apical zone of a *P. vulgaris* transgenic root (lower panel). * Indicates periclinal division, which may correspond to division of cortical initials. The corresponding differential interference contrast (DIC) image is shown in the upper panel. Information that can be extracted

from figure in A includes: (**B**) Number of cells with anticlinal, periclinal and oblique orientation. (**C**) Distance separating the cell plate from two contiguous cells. (**D**) Length of the forming cell plate, from which the stages of mitosis can be inferred using information from roots of model plants, previously characterized. (**E**) Comparative analysis of cell dimensions in different zones of the root, to identify transition zones as indicated by the dashed line in A. Student *t* test analysis confirmed that differences in cell length in E are statistically significant, but width data in graphs, **** indicate statistically different with $p < 0.00001$, respectively; ns, no significant difference ($p > 0.05$). Bars = 20 μm (**A**) and 5 μm (**B**–**D**).

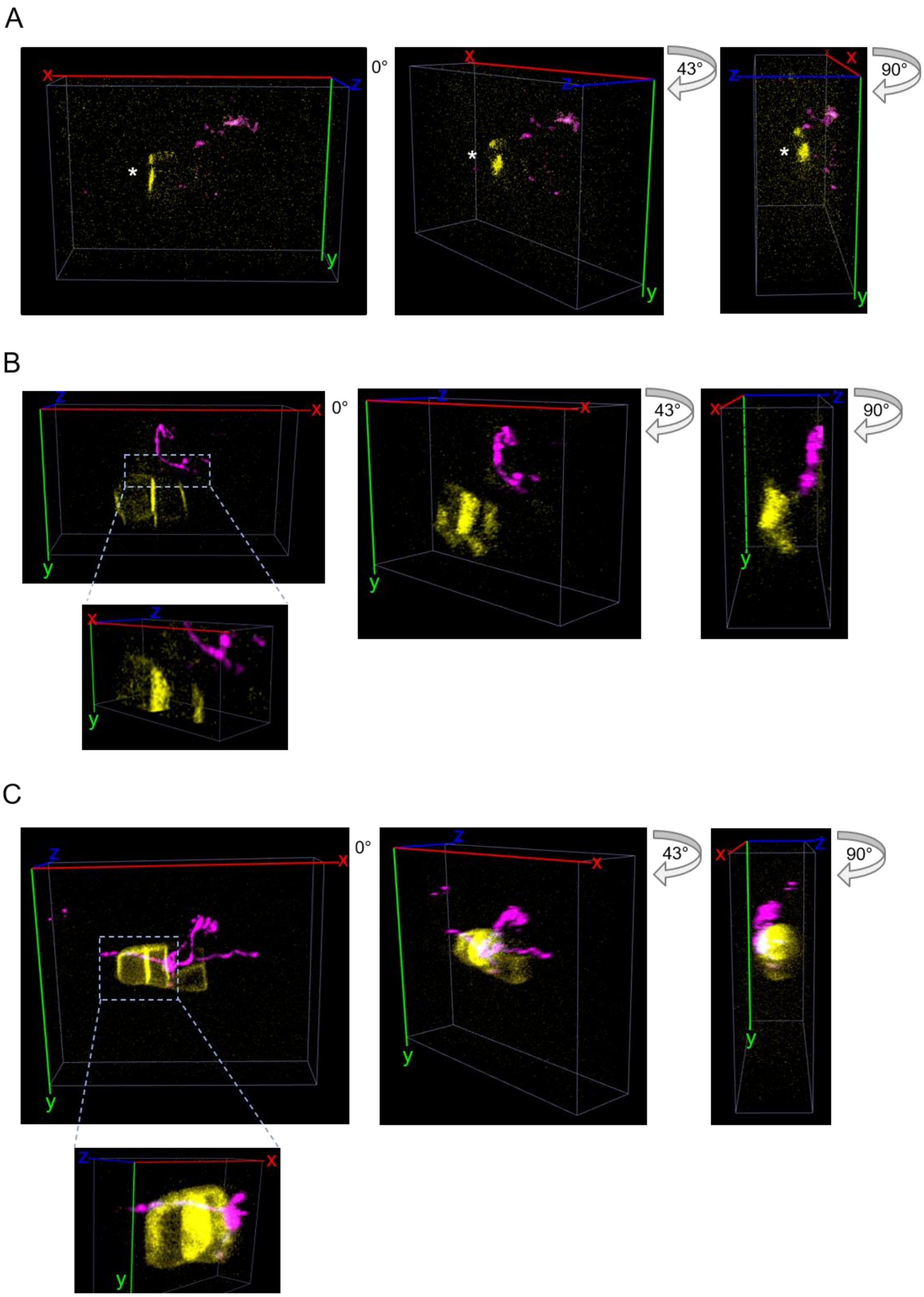
A
x
z
y
0°
43°
90°
*
B
x
z
y
0°
43°
90°
C
x
z
y
0°
43°
90°

**Supplementary Figure S6.** 3D reconstruction of the epidermal infection site illustrated in Figure 5B–D. Reconstruction was created using Vaa3D® software (3D Visualization-Assisted Analysis). (**A**) Stage II reconstructed from z-stacks = 54 μm, with a step size = 1.0 μm per slice. (**B**) Stage III, reconstructed from z-stacks = 52 μm, with a step size = 1.0 μm per slice. (**C**) Stage IV, reconstructed from z-stacks = 48 μm, with a step size = 1.0 μm per slice. The angles of rotation are indicated in each panel.

## **Supplementary Table 1.** Oligonucleotides used in this study

| **Primer name** | **Primer sequences** |
|---|---|
| pPvKNOLLE UP | 5'-CACCGGTTAAGTCTCAGGCTCTTTTAACAATG-3' |
| pPvKNOLLE LW | 5'-TTTGGGAACTTTGGATTGATAATGG-3' |
| PvKNOLLE-ATG | 5'-CACCATGAACGATCTAATGACCAAGTCCTTC-3' |
| PvKNOLLE stop | 5'-TCAAGAACTGCTTAAACTGGTGGC-3' |
| NLS-Turquoise UP | 5'-ATGCCAAAGAAGAAGAGGAAGGTTGTGAGCAAGGGCGAGGAG-3' |
| Turquoise LW | 5'CTACTTGTACAGCTCGTCCATGCC3' |
